# Assessing causal associations of blood counts and biochemical indicators with pulmonary arterial hypertension: a Mendelian randomization study and results from national health and nutrition examination survey 2003–2018

**DOI:** 10.3389/fendo.2024.1418835

**Published:** 2024-06-17

**Authors:** Zhekang Liu, Qingan Fu, Qingyun Yu, Xiaowei Ma, Renqiang Yang

**Affiliations:** ^1^ Cardiovascular Medicine Department, The Second Affiliated Hospital of Nanchang University, Nanchang, Jiangxi, China; ^2^ Rheumatology and Immunology Department, The Second Affiliated Hospital of Nanchang University, Nanchang, Jiangxi, China

**Keywords:** blood counts, biochemical indicators, pulmonary arterial hypertension, Mendelian randomization, causal association

## Abstract

**Background:**

Blood counts and biochemical markers are among the most common tests performed in hospitals and most readily accepted by patients, and are widely regarded as reliable biomarkers in the literature. The aim of this study was to assess the causal relationship between blood counts, biochemical indicators and pulmonary arterial hypertension (PAH).

**Methods:**

A two-sample Mendelian randomization (MR) analysis was performed to assess the causal relationship between blood counts and biochemical indicators with PAH. The genome-wide association study (GWAS) for blood counts and biochemical indicators were obtained from the UK Biobank (UKBB), while the GWAS for PAH were sourced from the FinnGen Biobank. Inverse variance weighting (IVW) was used as the primary analysis method, supplemented by three sensitivity analyses to assess the robustness of the results. And we conducted an observational study using data from National Health and Nutrition Examination Survey (NHANES) 2003–2018 to verify the relationship.

**Results:**

The MR analysis primarily using the IVW method revealed genetic variants of platelet count (OR=2.51, 95% CI 1.56-4.22, P<0.001), platelet crit(OR=1.87, 95% CI1.17-7.65, P=0.022), direct bilirubin (DBIL)(OR=1.71, 95%CI 1.18-2.47,P=0.004), insulin-like growth factor (IGF-1)(OR=0.51, 95% CI 0.27-0.96, P=0.038), Lipoprotein A (Lp(a))(OR=0.66, 95% CI 0.45-0.98, P=0.037) and total bilirubin (TBIL)(OR=0.51, 95% CI 0.27-0.96, P=0.038) were significantly associated with PAH. In NHANES, multivariate logistic regression analyses revealed a significant positive correlation between platelet count and volume and the risk of PAH, and a significant negative correlation between total bilirubin and PAH.

**Conclusion:**

Our study reveals a causal relationship between blood counts, biochemical indicators and pulmonary arterial hypertension. These findings offer novel insights into the etiology and pathological mechanisms of PAH, and emphasizes the important value of these markers as potential targets for the prevention and treatment of PAH.

## 1 Introduction

Pulmonary arterial hypertension (PAH) is classified as a rare yet life-threatening condition affecting the pulmonary arteries, with an estimated incidence of 10.6 cases per million adults in the United States (1). The initial manifestations of PAH can be quite indistinct, commonly presenting as exertional fatigue and shortness of breath. The disease is characterized by heightened pressures within the pulmonary artery, stemming from both constriction and structural changes in the pulmonary vessels. Despite recent advances and updates in the understanding of PAH treatments, the nonspecific nature of early symptoms and heavy dependence on invasive diagnostic methods such as right heart catheterization or complex imaging often result in delayed diagnosis and intervention. Finally, PAH can advance to terminal right heart failure or lead to mortality, with an approximate yearly death rate of 10% (1, 2). Therefore, there is an urgent need for a simple method to assist clinicians in the early identification of risk factors for PAH in their clinical practice, as well as to screen for the risk of PAH in high-risk populations, such as patients with connective tissue disease.

Blood counts and biochemical markers are among the most common tests performed in hospitals and most readily accepted by patients, and are widely regarded as reliable biomarkers in the literature (3, 4). Given their ease of administration, blood counts and biochemical markers have great potential to be a quick way to predict PAH risk, and similarly, a large number of studies have observed an association between some blood counts and biochemical markers and PAH. In a Mendelian randomization (MR) study, erythrocyte distribution width was identified as a predictor of PAH survival and is a clinically accessible and validated biomarker (5). Another study exploring prognostic markers of PAH showed that blood cell counts and biochemical markers such as platelets, bilirubin, and uric acid were strongly associated with the prognosis of PAH (6). However, some researchers have found different results that platelet distribution width and mean platelet volume can only reflect the degree of PAH condition, but not a prognostic predictor (7). These diverse findings highlight the potential significance of blood counts and biochemical markers in early PAH risk assessment, emphasizing the need for in-depth studies of their PAH predictive value.

MR is a novel epidemiological approach that uses genetic variation as an instrumental variable (IV) to proxy for exposure based on genome-wide association study (GWAS) data.MR methods minimize bias in results due to common environmental confounders and interference with reverse causality, and have been used in recent years primarily to infer causal relationships between exposures and outcomes (8). Compared with traditional clinical studies, MR studies have lower cost, time-consuming, larger data size and no ethical constraints, which can compensate for the shortcomings of traditional clinical studies in exploring genetic causality (9). The aim of this study was to investigate whether there is a causal relationship between 53 blood cell counts and biochemical markers and PAH using a two-sample MR approach, and to provide more evidence for the use of blood cell counts and biochemical markers in early PAH risk prediction in clinical practice.

## 2 Methods

### 2.1 Two-sample MR

**Figure 1:**
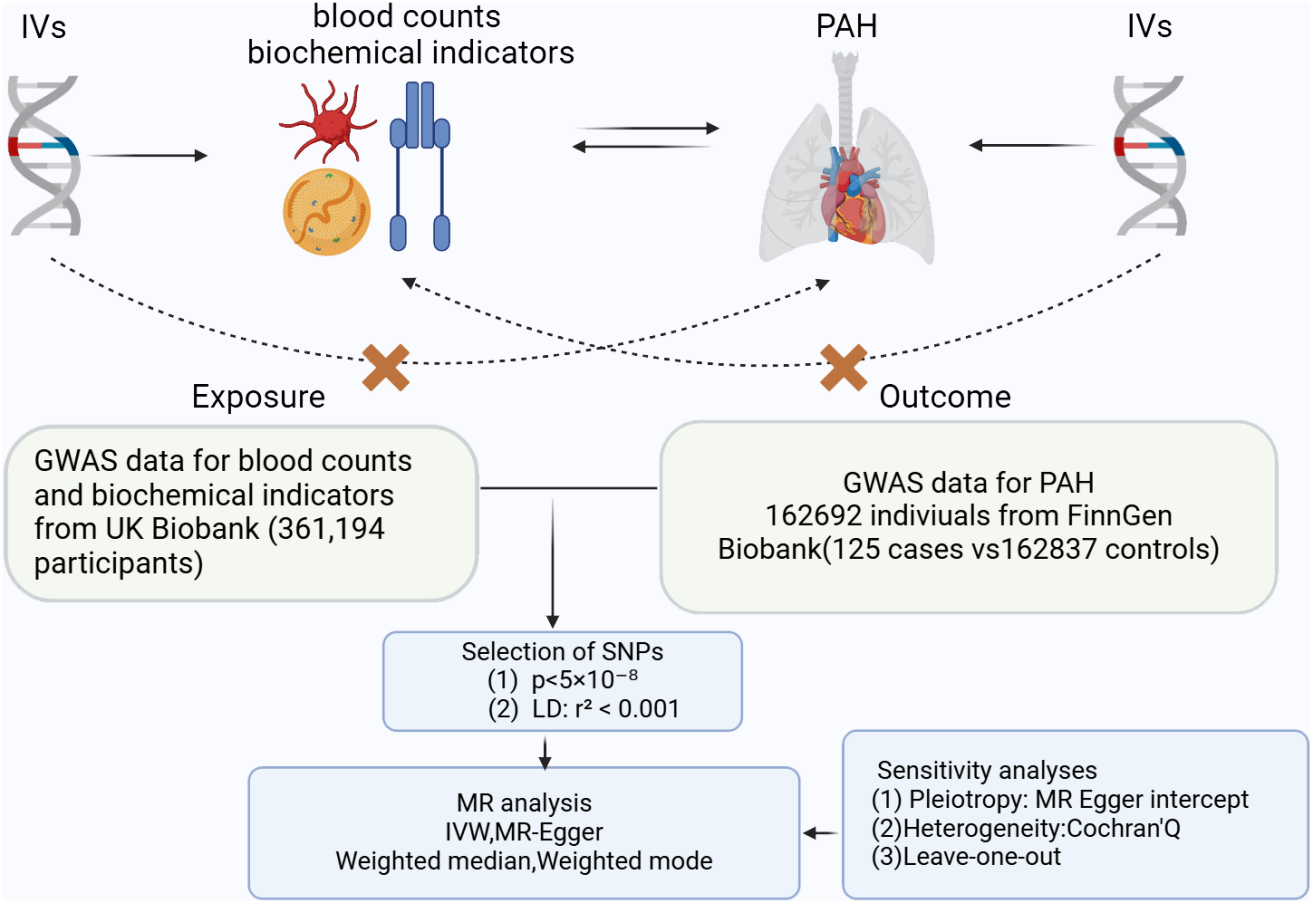
Three key principles of bidirectional Mendelian randomization study design. IVs, instrumental variables. PAH, pulmonary arterial hypertension.

A two-sample MR Study was conducted to assess whether there is a causal relationship between blood counts, biochemical indicators and PAH. A range of SNPs was utilized as the instrumental variables for this analysis. The foundational principles of MR necessitate that: firstly, the instrumental variables must have a robust association with the exposure; secondly, they should not be correlated with confounders; and thirdly, the selected IVs do not have a direct association with the outcome (**Figure 1**).

### 2.2 Data sources

The GWAS data is a genome-wide association study of the largest European population and provides the basis for use in studies of blood counts, biochemical indicators and PAH. Summary-level statistical data for PAH were obtained from the FinnGen GWAS results (10). The summary statistics for blood counts, biochemical indicators and PAH was obtained from the UK Biobank. The GWAS data used in this study is given in **Supplementary Table 1**. The National Health and Nutrition Examination Survey (NHANES) is a program designed to assess the health and nutritional status of populations in the U.S. The program has been formally approved by local ethical review boards, and NHANES has obtained informed written consent from all individuals participating in the study (11). The NHANES dataset analyzed in this study from 2003 to 2018 was downloaded from the official NHANES website. (https://www.cdc.gov/nchs/nhanes/index.html). Risk factors commonly associated with pulmonary arterial hypertension (PAH) include systemic hypertension, diabetes, uric acid level, age, insulin status, obesity, thyroid problems, and hormone therapy (12). Based on this research, we isolated a subset of the 2003-2018 NHANES data that contained all PAH high-risk populations. Specifically, we classified male subjects with two or more of these risk factors and female subjects with three or more (inclusive of hormone therapy) as individuals possessing combined risk factors for PAH, referred to as “subjects with combined risk factors for PAH (13). Logistic performed simultaneous multivariate analyses with multivariate models with covariates of sex, age(year), Hemoglobin (HB), diastolic blood pressure (DBP), systolic blood pressure (SBP). Raw data excluded missing more than 10% of the variables, and those less than 10% were filled in using median filler. Participants devoid of any risk factors for PAH were incorporated into the no-risk factor or control group, raw data excluded missing more than 10% of the variables, and those less than 10% were filled in using median filler, and a final total of 453 patients were included. The data are detailed in **Supplementary Table 2**.

### 2.3 Selection of IVs

For genetical instruments of blood counts and biochemical indicators on PAH, we used single nucleotide polymorphisms (SNPs) that were strongly associated with each exposure (p < 5 × 10^−8^) as instruments. In reverse analysis, the standard for P has been relaxed (p < 5 × 10^−6^) to include more SNPs. We screened LD independent SNPs based on R2 = 0.001, window size = 10,000 kb. SNPs overlapped with the risk of outcome (p < 5 × 10^−6)^ were eliminated to prevent potential multiple effects.

### 2.4 Statistical analyses

The “TwoSampleMR” packages of the R software (version 4.2.2) were used to perform MR analysis. Several MR methods were used to infer causal relationships blood counts, biochemical indicators and PAH. These methods included inverse variance weighting (IVW), MR-Egger, median weighting, and weighted mode and IVW method is regarded as the most important method in the study (14–17). To assess the association between blood counts, biochemical indicators and PAH. MR analysis was performed. IVW was considered to be the primary MR method, and p < 0.05 and consistent direction of the four MR methods were considered significant. Multivariate Mendelian randomization(MVMR) was used to explore the independent effect of exposure on outcome (18). To ensure the accuracy of the results, the palindromic SNPs were removed from the analysis. Regarding the baseline NHANES data, we expressed continuous variables as medians (interquartile range [IQR]) and tested them using the Mann-Whitney U, whereas the chi-square test was used to compare categorical variables presented as frequencies and percentages (%). In addition, the correlation between total bilirubin, platelet count, platelet volume and PAH risk was explored by means of univariate and multivariate logistic regression with calculation of odds ratios (OR) and 95% confidence intervals (95% CI). Multivariate logistic regression adjusted for sex, age, HB, DBP, SBP based on univariate logistic regression, R (version 4.2.2) was used to perform the statistical analysis of the NHANES data, and all tests were two-sided with a significance level of 0.05.

### 2.5 Sensitivity analyses in MR

To assess if a single SNP could significantly influence causality, we conducted ‘leave-one-out’ sensitivity tests (**Supplementary Figure 2**). For heterogeneity measurement, we applied Cochran’s Q statistic. Where heterogeneity was present, we opted for the random effects IVW (multiplicative random effects) method for more conservative and robust estimations (**Supplementary Table 2**).

## 3 Results

### 3.1 Univariate MR analysis

SNPs linked to these conditions were chosen as instrumental variables, adhering to predefined quality standards. Each SNP was linked to the exposure without any direct association with the outcome, ensuring their suitability as IVs. The F-statistics of these SNPs were above the threshold of 10 (19), indicating that they strongly represented these phenotypes in the MR analysis. IVs used in the MR Analysis are available in the **Supplementary Table 2**.

**Figure 2:**
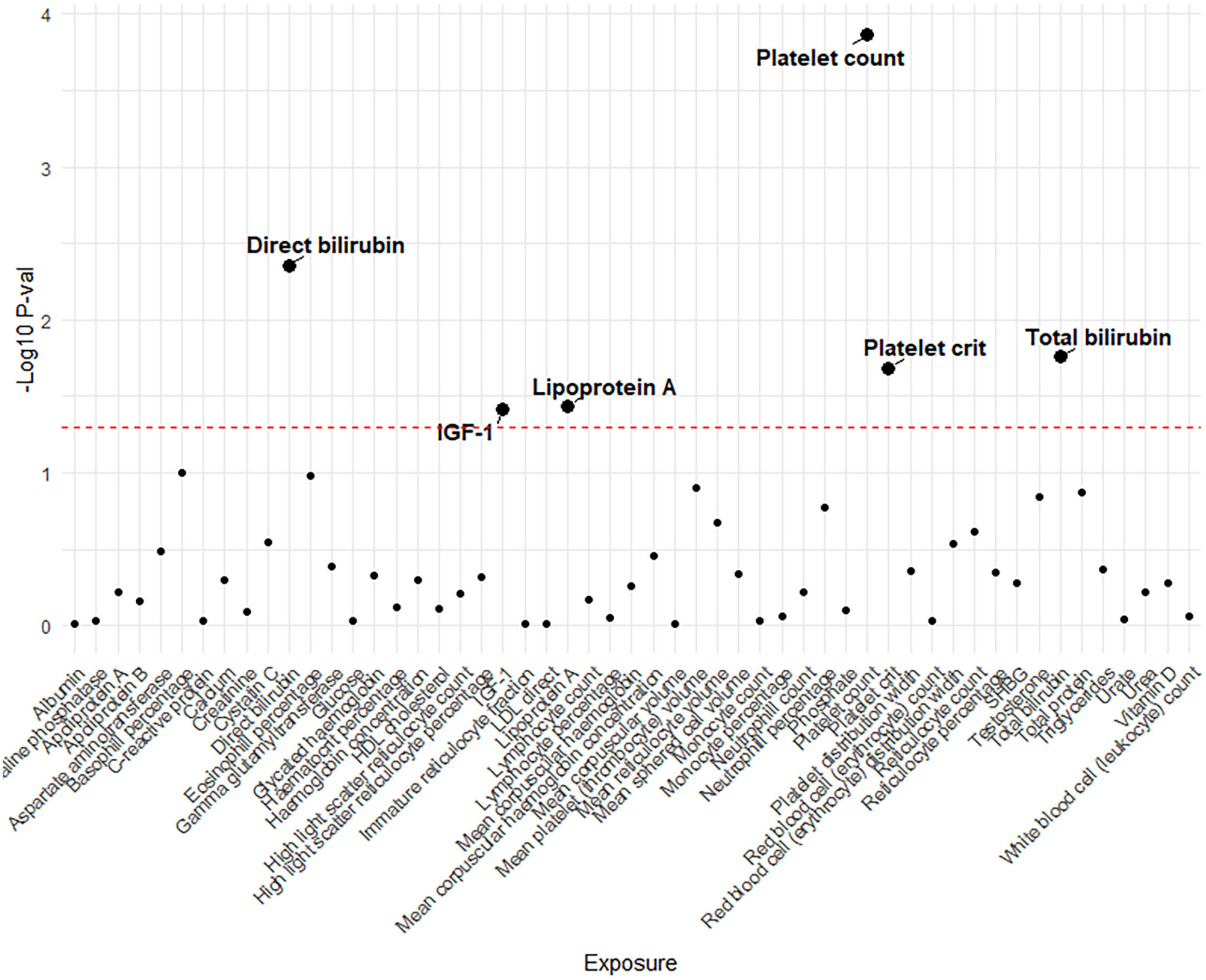
The p-value distribution of associations between 53 blood counts and biochemical indicators and pulmonary arterial hypertension in the Mendelian randomization analysis. The dashed line represents the threshold of suggestive level of significance, set at p= 0.05, (-Log10 p -val = 1.3).

**Figure 3:**
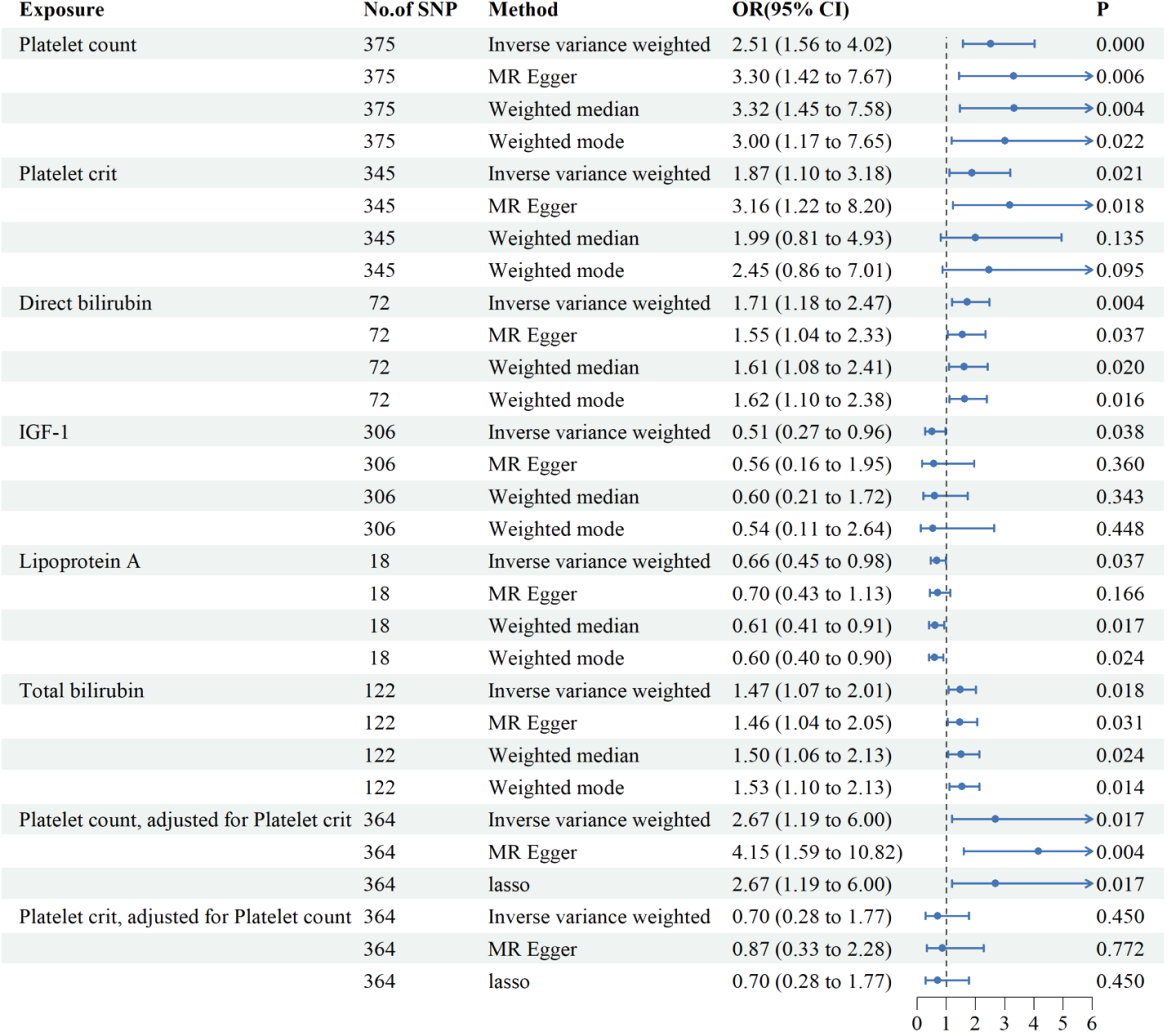
Associations between genetically predicted 6 blood counts and biochemical indicators and pulmonary arterial hypertension examined by four MR methods.MR, Mendelian randomization; IVW, inverse-variance weighted, CI, confidence interval.

**Figure 4:**
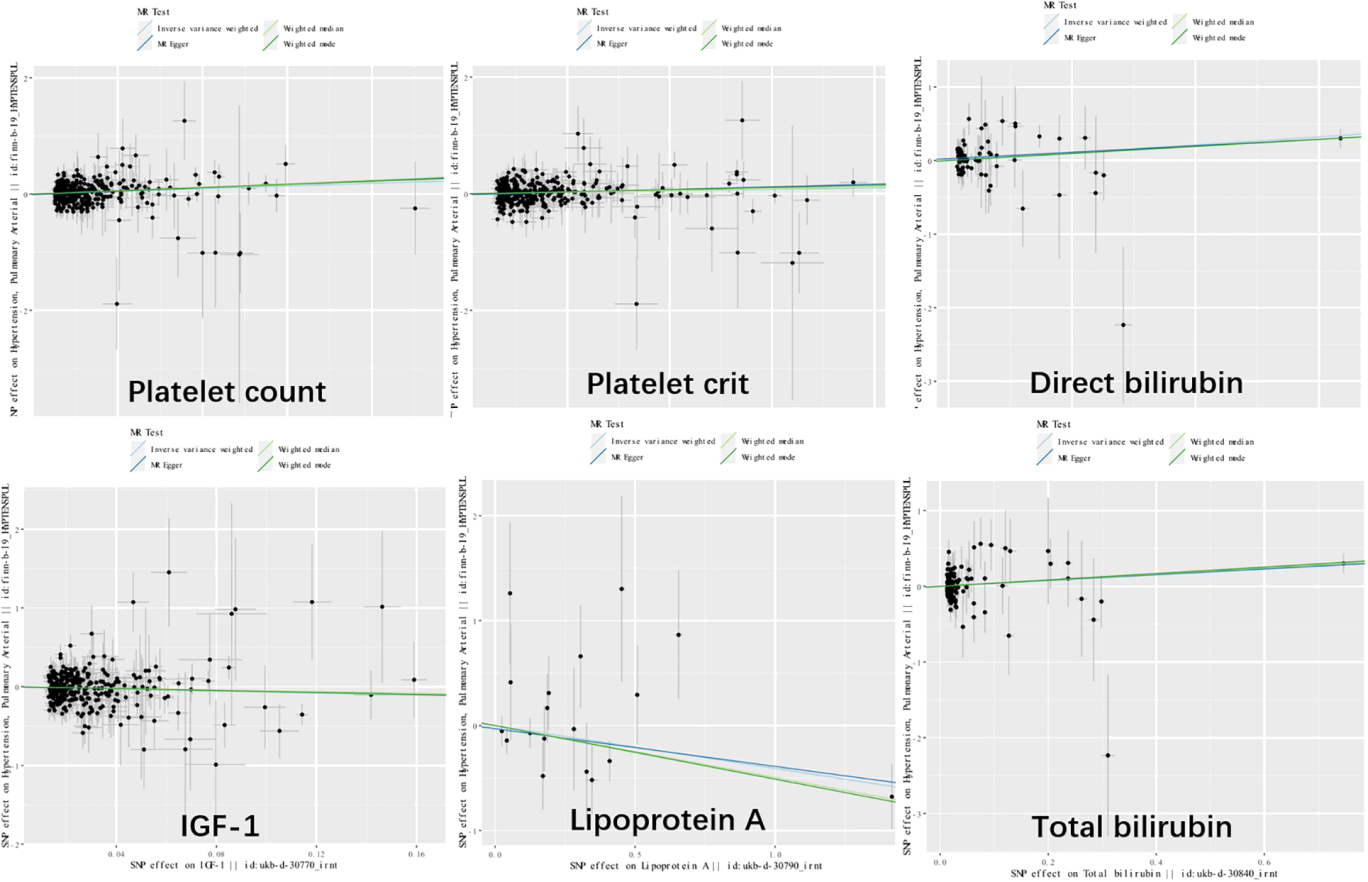
Scatter plot showing the causal effects of 7 blood counts and biochemical indicators on pulmonary arterial hypertension. SNP, single nucleotide polymorphism.

Genetic variants of platelet count (OR=2.51, 95% CI 1.56-4.22, P<0.001), platelet crit(OR=1.87, 95% CI1.17-7.65, P=0.022), DBIL(OR=1.71, 95%CI1.18-2.47,P=0.004), IGF-1(OR=0.51, 95% CI0.27-0.96, P=0.038), Lp(a) (OR=0.66, 95% CI0.45-0.98, P=0.037) and TBIL(OR=0.51, 95% CI0.27-0.96, P=0.038) were associated with PAH. (**Figures 2**, **3**). **Figure 4** presents a scatter plot that delineates the inferred causal linkages between six blood counts and biochemical indicators and PAH.

We also analyzed potential heterogeneity (**Supplementary Table 2**). Based on MR-Egger’s examination of the intercept term, the causal analysis did not show significant evidence of horizontal pleiotropy (**Supplementary Table 2**).

### 3.2 Multivariate and reverse MR analysis

Since platelet count and platelet crit interacted with each other, we further investigated the role of each independently on PAH by MVMR. Platelet count elevated the risk of developing PAH by 167% (OR=2.67,95%CI1.19-6.00, *P*=0.017) after adjustment for platelet crit and the effect of platelet crit is no longer present with adjustment for platelet count. Multivariate MR suggests platelet count is an important risk factor for PAH.

Meanwhile, we explored reverse causality using PAH as exposure and 53 blood counts and biochemical indicators as outcomes. The results indicated a suggestive causal relationship between PAH and basophil percentage, albumin, Lp(a), urate (**Supplementary Figure 1**). The OR interval is too small to be statistically significant, while suggesting that our MR study is unlikely to be confounded by reverse causality.

### 3.3 In NHANES

**Supplementary Table 2** shows the baseline data for the PAH high risk population and the general control population, we observed that the PAH high risk population had significantly higher levels of leukocyte counts/age/BMI/platelet volume and uric acid and were more likely to be male/hypertensive and diabetic, while haemoglobin levels/platelet counts were significantly lower in comparison to healthy individuals. Where the median and range of total bilirubin/platelet count and volume were 11.970 (8.550 - 13.680), 234.000 (196.000 - 282.000) and 8.200 (7.600 - 8.800) respectively. Subsequently, we explored the relationship between total bilirubin/platelet count and volume and the risk of developing PAH using univariate and multivariate logistic regressions. In univariate logistic regressions, platelet count was significantly negatively correlated with the risk of PAH, while the opposite was true for platelet volume, while no relationship was observed between total bilirubin and PAH. After adjusting for sex, age, hemoglobin, systolic, and diastolic blood pressure, multivariate logistic regression analyses revealed a significant positive correlation between platelet count and volume and the risk of PAH, and a significant negative correlation between total bilirubin and PAH **Table 1**. The results suggest that platelet-related markers are important risk factors for PAH, which is consistent with the results of our MR analyses, although total bilirubin may be protective against PAH development in the NHANES database population.

**Table 1:** Odd ratios (95% confidence intervals) for total bilirubin, platelet count, platelet volume and the risk of PAH.

Name	N	OR	95%CI	P-value	N_adjusted	OR_adjusted	95%CI_adjusted	P-value_adjusted
Total bilirubin	15835	0.997	[0.99,1.005]	0.512	15835	0.988	[0.979,0.997]	0.009
Platelet count	15835	0.999	[0.998,1.0]	0.002	15835	1.001	[1.001,1.002]	0.000
Platelet volume	15835	1.065	[1.019,1.113]	0.005	15835	1.081	[1.031,1.133]	0.001

Univariate and multivariate logistic regression models were used to estimate OR and 95% CI. adjusted for sex, age, hemoglobin, systolic and diastolic blood pressure. OR, odd ratios; CI, confidence intervals.

## 4 Discussion

zIn this study, MR statistical method was used to reveal the association between 53 blood count, biochemical indicators and PAH, and the results revealed that 6 of them were causally related to PAH(P<0.05). Among them, platelet count, platelet crit, direct bilirubin, total bilirubin raised the risk of developing PAH. In addition, IGF-1, Lp(a) were protective factors for PAH.

### 4.1 Platelet

In the present study, we found significant positive causal effects of both platelet count and platelet pressure on PAH in univariate MR, and platelet count was still significantly positively causally associated with increased risk of PAH in multivariate-adjusted MR results, however, similar results were not demonstrated in reverse MR. This suggests that platelet crit may causally contribute to the outcome leading to an increased risk through a rise in platelet count, and that platelet count is a significant risk factor and predictor of PAH and is not confounded by reverse causality effects, which provides novel evidence at the causal level of traditional clinical studies in this area. Aligning with prior research, our results underscore the significant role of platelets in the progression of PAH through various mechanisms.

Platelets are a key substance that plays a role mainly in hemostasis in the body and are present in the blood as small discoidal cell fragments (20). Existing studies suggest that it is mainly the increased number of activated platelets and the upregulation of the expression of various platelet-derived factors that in turn contribute to the progression of PAH (21, 22). First, platelets secrete a variety of growth factors that play an important role in the pathologic changes of PAH, such as platelet-derived factor (PDGF) and platelet transforming growth factor β1 (TGF-β1).PDGF is a platelet-derived chemotactic substance and mitogen, and PDGF has been observed to promote the excessive proliferation and contraction of vascular smooth muscle cells in animal models of PAH, in particular, PDGF-BB (an active factor of the PDGF family), but researchers have found that Ligustrazine inhibits PDGF-BB-induced proliferation and contraction of pulmonary artery smooth muscle cells by modulating the PI3K/AKT pathway as a treatment for PAH (23, 24). It has also been found that PDGF can be degraded in alveolar macrophages by regnase-1 to alleviate the progression of PAH, demonstrating the critical role of PDGF in PAH, and the use of chlordiazepoxide and the overexpression of regnase-1 have been recognized as potential therapeutic approaches for the treatment of PAH (25). TGF-β1 is a cytokine involved in vascular remodeling and pulmonary fibrosis and is the main cytokine secreted by activated platelets in patients with PAH, with platelets secreting 45% of the total amount of TGF-β1 in the internal environment. TGF-β1 significantly upregulates aerobic glycolytic processes in pulmonary arterial smooth muscle cells, which directly contributes to vascular remodeling and metabolic reprogramming, ultimately leading to thickening of small pulmonary arteries and formation of plexiform lesions. In animal studies, TGF-β1-depleted mice were able to resist hypoxia-induced PAH to a certain extent, whereas in human studies, platelet glycolysis levels were found to correlate with the severity of symptoms in patients with PAH (26, 27). Next, platelet-derived microparticles (PDMP) are microsomes released by platelets upon stimulation, which contribute to PAH development by promoting inflammation and thrombosis (28). This may be mediated by PDMP overexpression of CD40 ligand as well as the procoagulant phosphatidylserine, and PDMP also enhances vasoconstriction and cell proliferation via thrombin (29, 30). Recently researchers have identified platelet Toll-like receptor 4 (TLR4) as a key factor mediating immune and inflammatory responses in PAH, and in a hypoxia-induced model, specific deletion of TLR4 demonstrated protective effects against PAH, and inhibition of TLR4-specific ligands or stimulation of ligand etiology may be a viable treatment for PAH (31). It has also been shown that the gradual hypoxia caused by the early onset of PAH will further lead to the activation of platelets, forming a vicious circle that again exacerbates the condition of patients with PAH, while the depletion of platelets is able to reduce the generation of chemokines, such as CXCL4 and CCL5, and slow down the progression of the disease (32). In addition, platelets may also be involved in the pathology of PAH through delayed adaptation to microvascular shear, which may be due to platelet adhesion factor-1 instability (33). However, a recent study have noted lower platelet counts in patients with PAH (34), which may be related to more thrombosis in patients with PAH leading to platelet depletion (35)and PAH is associated with a chronic hypoxic state, which may affect bone marrow function and lead to decreased platelet production (36). It has also been noted that splenomegaly occurs in 52 ~ 63% of PAH patients (37), which may increase filtration and consumption of platelets by the spleen. Our MR study effectively avoids the reverse causality. In conclusion, platelet count is an extremely important and accessible predictor of PAH, which can greatly assist clinicians in the early identification of PAH risk and allow high-risk patients to be diagnosed and treated as soon as possible.

### 4.2 Bilirubin

Our study identified a suggestive positive causal relationship between DBIL, TBIL and PAH. Many recent clinical studies have shown that bilirubin is a strong and readily available predictor of poor prognosis in PAH (38–40), which is consistent with the findings of our MR study. The mechanism of the relationship between elevated bilirubin and adverse PAH outcomes remains unclear (39). Previous studies have shown that the oxidative stress pathway is involved in the pathogenesis of PAH and that the oxidative stress pathway may be an important target for PAH treatment (41–43). It has also been noted that bilirubin is unusually sensitive to hemodynamic alterations and is a strong predictor of poor prognosis, even after adjusting for age-sex (38, 44). It has also been suggested that PAH may cause right heart failure, which in turn causes congestive liver disease, resulting in elevated bilirubin (38). Our study explains the importance of PAH testing bilirubin levels from a genetic perspective and providing a substantial addition to clinical research. Furthermore, our MR analysis effectively mitigates the concerns of reverse causality.

### 4.3 IGF-1

The research conducted demonstrated that IGF-1 has a protective effect against PAH. IGF-1 is mainly produced by the liver and circulates in the bloodstream (45), has been the focus of research due to its wide range of physiological roles and therapeutic potential. Previous study have shown that IGF-1 promotes the proliferation and migration of vascular smooth muscle cells and endothelial cells, contributing to the maintenance of vascular structure and function (45–48). In PAH, this can help mitigate pathological vascular remodeling, including intimal thickening and vascular wall hypertrophy. In addition, IGF-1 has anti-inflammatory properties, reducing the activation of inflammatory cells and the release of inflammatory cytokines (46), which may help alleviate the inflammatory response in PAH. IGF-1 can also activate various cell signaling pathways, protecting cells from apoptosis and other forms of cell damage, crucial for maintaining the survival and function of vascular cells (49). Previous reports on the relationship between IGF-1 and blood pressure regulation in hypertensive disorders are conflicting, with some studies indicating higher levels of IGF-1 in people with hypertension (50–52), yet several large cross-sectional studies demonstrating a inverse relationship (53–55). Our study suggests that IGF-1 may be a potential therapeutic target for PAH, and further research is needed to validate these causal effects.

### 4.4 Lipoprotein A

This study also reveals the protective effect of Lp(a) against PAH. Lp(a) is an almost strictly genetically controlled lipoprotein, and more than 90% of the variation in concentration can have a genetic explanation (56). It has been suggested that Lp(a) interferes with many of the key reactions to hemolysis and fibrinolysis *in vitro (* 57, 58), and that Lp(a) contributes to smooth muscle accretion by impairing transforming growth factor B activation through downregulation of fibrinogenesis (59), which might help to maintain the structure and function of the pulmonary arteries. Previous studies have concluded that Lp(a) significantly increases the probability of developing coronary heart disease, whereas patients with coronary heart disease tend to have a lower risk of PAH, the protective effect of Lp(a) on PAH may be related to this association (60–62). Our study provides a new insight into Lp(a) in PAH and more clinical and basic studies are needed to further validate this finding.

### 4.5 Strengths and limitations

This study exhibits several noteworthy strengths. Firstly, the use of two-sample MR adds a level of rigor in establishing causality, not just correlation. Secondly, utilizing data from large-scale GWAS studies like FinnGen Biobank and the UK Biobank lends credibility and robustness to the findings. Thirdly, the inclusion of multiple statistical methods, sensitivity analyses, and adjustment for various confounders provides a thorough exploration of the data. Last, we validated the results using clinical data from the US population, further increasing the robustness and population applicability of the results.

Our study also has some limitations. Firstly, these findings based on European populations might not be generalizable to other ethnicities. Second, Lack of demographic data precluded subgroup analysis. Third, our study focused more on screening for potential causality, with a significance level of P < 0.05, and some positive results did not continue after correction for multiple comparisons. Last, the treatment of PAH may affect the accuracy of the results to some extent. Some PAH medications, particularly those metabolised by the liver, can affect liver function and thus may affect bilirubin levels, such as prostacyclin analogues and endothelin receptor antagonists (63). Drugs such as endothelin receptor antagonists (e.g. bosentan) have also been reported to cause thrombocytopenia in some patients (64). These studies further point to the importance of monitoring bilirubin, platelets, and other markers in patients with PAH.

### 4.6 Conclusion

Our findings offer robust causal evidence to researchers in PAH, demonstrating a close correlation between certain blood counts and biochemical markers with the onset and progression of PAH. This emphasizes the significant value of these markers as potential targets for the prevention and treatment of PAH. Future large-scale, prospective studies are needed to validate our findings, particularly to delve deeper into the potential biological mechanisms linking these hematological and biochemical markers with PAH risk.

## Data availability statement

The original contributions presented in the study are included in the article/**Supplementary Material**. Further inquiries can be directed to the corresponding author.

## Ethics statement

This study utilized data from participant studies that were publicly available and approved by an ethical standards committee for human experimentation. No additional ethical approval was deemed necessary for this particular study.

## Author contributions

ZL: Formal analysis, Resources, Software, Supervision, Validation, Writing – original draft. QF: Writing – original draft. QY: Validation, Conceptualization, Writing – review & editing. XM: Software, Writing – review & editing. RY: Funding acquisition, Writing – review & editing, Software.
