# Supplementary material for: Assessing causal associations of blood counts and biochemical indicators with pulmonary arterial hypertension: a Mendelian randomization study and results from national health and nutrition examination survey 2003–2018
Source: Front Endocrinol (Lausanne). 2024 Jun 17;15:1418835. doi: 10.3389/fendo.2024.1418835 (PMC11215008; doi:10.3389/fendo.2024.1418835)
Supplement: Supplementary file 1 [file DataSheet_1.docx]

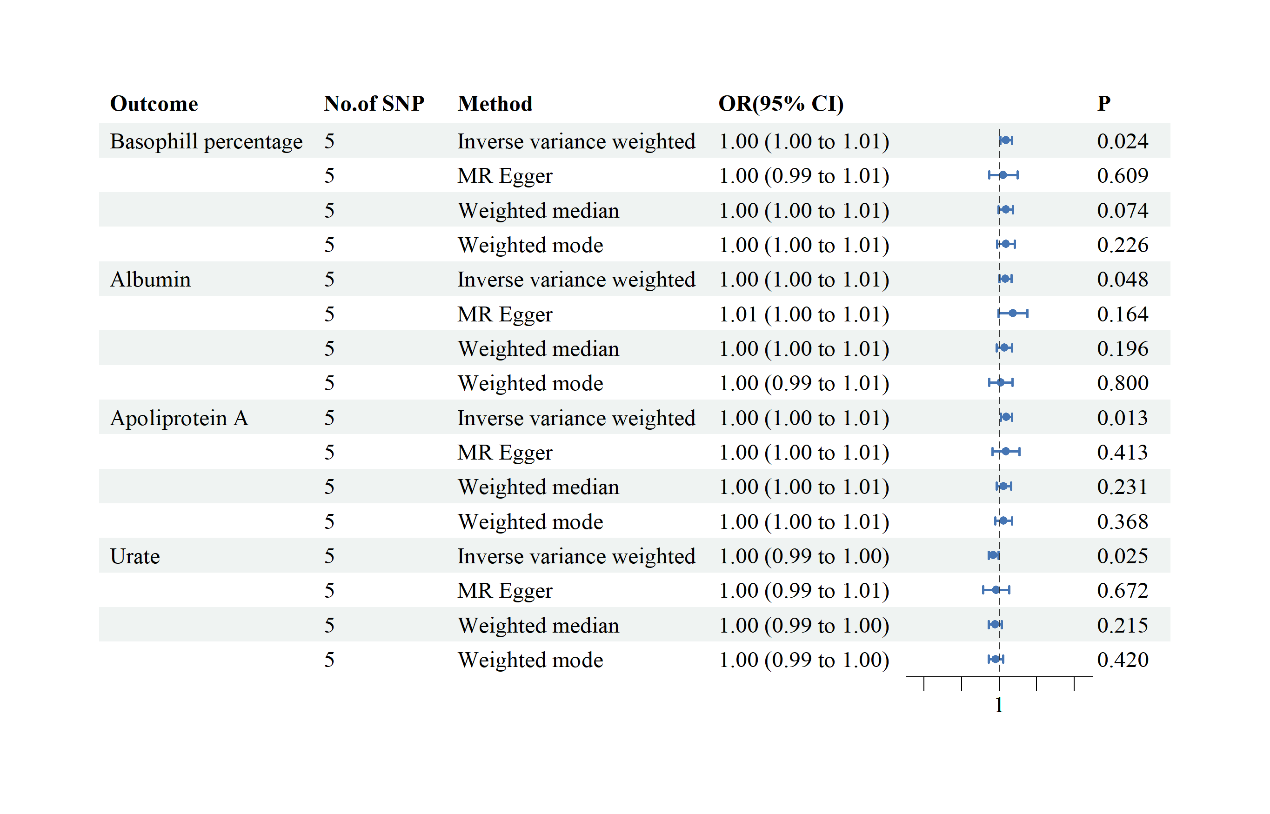


**Supplementary Figure1. Reverse MR analyses results**

**
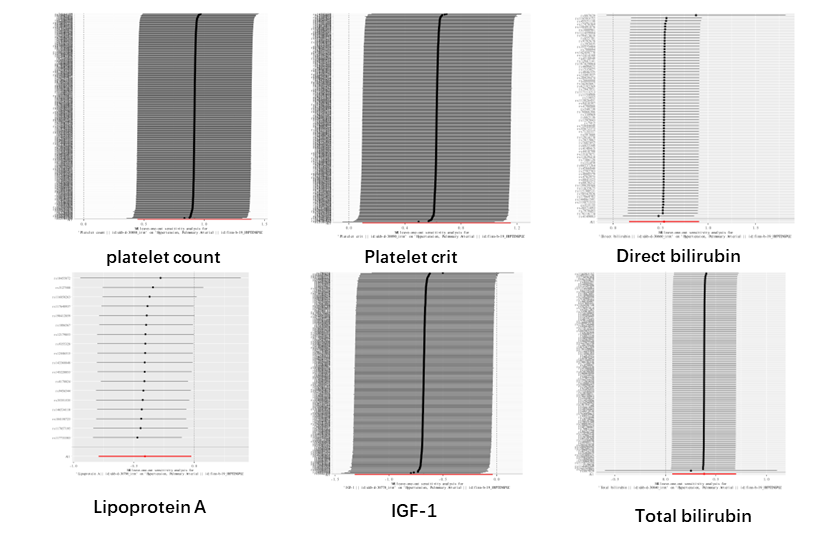
Supplementary Figure2. leave-one-out plots**

**Supplementary Table 1.** Information of GWAS summary datasets used in MR analyses. The GWAS from the Finnish Biobank source representing pulmonary hypertension had 125 cases and 162,837 controls. The GWAS for blood counts and biochemical indices from the UK Biobank source had 361,194 subjects

| Traits | GWAS.ID |
| --- | --- |
| Pulmonary arterial hypertension | finn-b-I9_HYPTENSPUL |
| Albumin | ukb-d-30600_irnt |
| Alkaline phosphatase | ukb-d-30610_irnt |
| Apoliprotein A | ukb-d-30630_irnt |
| Apoliprotein B | ukb-d-30640_irnt |
| Aspartate aminotransferase | ukb-d-30650_irnt |
| Basophill percentage | ukb-d-30220_irnt |
| Calcium | ukb-d-30680_irnt |
| Cholesterol | ukb-d-30690_irnt |
| C-reactive protein | ukb-d-30710_irnt |
| Creatinine | ukb-d-30700_irnt |
| Cystatin C | ukb-d-30720_irnt |
| Direct bilirubin | ukb-d-30660_irnt |
| Eosinophill percentage | ukb-d-30210_irnt |
| Gamma glutamyltransferase | ukb-d-30730_irnt |
| Glucose | ukb-d-30740_irnt |
| Glycated haemoglobin | ukb-d-30750_irnt |
| Haematocrit percentage | ukb-d-30030_irnt |
| Haemoglobin concentration | ukb-d-30020_irnt |
| HDL cholesterol | ukb-d-30760_irnt |
| High light scatter reticulocyte count | ukb-d-30300_irnt |
| High light scatter reticulocyte percentage | ukb-d-30290_irnt |
| IGF-1 | ukb-d-30770_irnt |
| Immature reticulocyte fraction | ukb-d-30280_irnt |
| LDL direct | ukb-d-30780_irnt |
| Lipoprotein A | ukb-d-30790_irnt |
| Lymphocyte count | ukb-d-30120_irnt |
| Lymphocyte percentage | ukb-d-30180_irnt |
| Mean corpuscular haemoglobin | ukb-d-30050_irnt |
| Mean corpuscular haemoglobin concentration | ukb-d-30060_irnt |
| Mean corpuscular volume | ukb-d-30040_irnt |
| Mean platelet (thrombocyte) volume | ukb-d-30100_irnt |
| Mean reticulocyte volume | ukb-d-30260_irnt |
| Mean sphered cell volume | ukb-d-30270_irnt |
| Monocyte count | ukb-d-30130_irnt |
| Monocyte percentage | ukb-d-30190_irnt |
| Neutrophill count | ukb-d-30140_irnt |
| Neutrophill percentage | ukb-d-30200_irnt |
| Phosphate | ukb-d-30810_irnt |
| Platelet count | ukb-d-30080_irnt |
| Platelet crit | ukb-d-30090_irnt |
| Platelet distribution width | ukb-d-30110_irnt |
| Red blood cell (erythrocyte) count | ukb-d-30010_irnt |
| Red blood cell (erythrocyte) distribution width | ukb-d-30070_irnt |
| Reticulocyte count | ukb-d-30250_irnt |
| Reticulocyte percentage | ukb-d-30240_irnt |
| SHBG | ukb-d-30830_irnt |
| Testosterone | ukb-d-30850_irnt |
| Total bilirubin | ukb-d-30840_irnt |
| Total protein | ukb-d-30860_irnt |
| Urate | ukb-d-30880_irnt |
| Urea | ukb-d-30670_irnt |
| Vitamin D | ukb-d-30890_irnt |
| White blood cell (leukocyte) count | ukb-d-30000_irnt |
| Abbreviation: GWAS: genome-wide association study; ID: Identification; MR: Mendelian randomization; ukb: UK Biobank; HDL: High-density lipoprotein; IGF-1: Insulin-like growth factor 1; LDL: Low-density lipoprotein; SHBG: Sex hormone-binding globulin.  **Supplementary Table 2 Heterogeneity and pleiotropy tests for the associations between blood counts, biochemical indicators and pulmonary arterial hypertension**   \| MR analysis \| Heterogeneity test \| \| Pleiotropy test \| \| \| \| --- \| --- \| --- \| --- \| --- \| --- \| \| Q \| Q-pval \| Egger_intercept \| se \| p \| \| platelet count \| 314.795 \| 0.987 \| -0.010 \| 0.032 \| 0.439 \| \| platelet crit \| 338.810 \| 0.554 \| -0.019 \| 0.015 \| 0.194 \| \| direct bilirubin \| 53.846 \| 0.924 \| 0.025 \| 0.023 \| 0.265 \| \| IGF-1 \| 323.373 \| 0.213 \| -0.002 \| 0.017 \| 0.867 \| \| Lipoprotein A \| 21.136 \| 0.173 \| -0.029 \| 0.086 \| 0.732 \| \| total bilirubin \| 97.368 \| 0.936 \| 0.001 \| 0.016 \| 0.732 \| | |
